# Analytical Method for the Simultaneous Determination of Albendazole and Metabolites Using HPLC-PDA: A Validation Study

**DOI:** 10.3390/molecules30092039

**Published:** 2025-05-03

**Authors:** E. Milena Vázquez, Beatriz Romero, Ana M. Sahagún, Cristina López, Raúl de la Puente, José M. Rodríguez, Nélida Fernández, M. José Diez, Raquel Díez

**Affiliations:** Pharmacology, Department of Biomedical Sciences, Veterinary Faculty, Institute of Biomedicine (IBIOMED), University of Leon, 24071 Leon, Spain; evaza@unileon.es (E.M.V.); amsahp@unileon.es (A.M.S.); clopcd@unileon.es (C.L.); rpueg@unileon.es (R.d.l.P.); jmrodl@unileon.es (J.M.R.); mnferm@unileon.es (N.F.); mjdiel@unileon.es (M.J.D.); rdielz@unileon.es (R.D.)

**Keywords:** albendazole, albendazole sulfoxide, albendazole sulfone, EMA guideline, HPLC, validation

## Abstract

Albendazole is an anthelmintic drug commonly used in animals and humans against nematodes. A sensitive, accurate, precise, and time-saving high-performance liquid chromatography (HPLC) method for the simultaneous determination of albendazole and metabolites (albendazole sulfoxide and albendazole sulfone) in cattle plasma has been developed and validated. A solid-phase extraction (SPE) was carried out. Separation was performed with an XBridge^®^ C18 column (4.6 mm × 250 mm, 5 µm) with gradient elution of acetonitrile:ammonium acetate buffer 0.025 M with pH adjusted to 6.6. The flow rate was 1.2 mL/min, and the PDA detector was set at 292 nm. Calibration curves were linear in the range from 0.025 to 2.0 µg/mL for the three compounds evaluated, with correlation coefficients ≥ 0.99. For the lower limit of quantification (LLOQ), within- and between-run precision and accuracy were satisfactory, with coefficients of variation (CV) ≤ 15.1% and deviations ≤ 117.7%, respectively. The method fulfilled all validation criteria established by the European Medicines Agency guideline (EMA/CHMP/ICH/172948/2019).

## 1. Introduction

Helminth infections are responsible for some of the most important diseases worldwide in animals. They continue to pose a major threat to livestock farming and may cause great economic losses due to reduced weight gain and poor feed conversion in animals. In addition, they prevent welfare from being ensured. Thus, they have an enormous importance, and effective control of parasites is essential for the profitability of livestock farms [1].

This control is mainly based on the use of antiparasitic drugs, but the inappropriate and intensive use of these compounds may lead to increased risk of anthelmintic resistance, which has become a major issue globally [2]. However, the high economic costs associated with the development of new anthelmintic drugs with different mechanisms of action, and the low number of new drugs that have come on the market in the last few decades make it unlikely that we will enter a new phase in the development of novel anthelmintic compounds [3]. Consequently, the available pharmacological groups should be employed in a much more efficient and rational way.

Benzimidazoles are among the most commonly used anthelmintics in ruminant species, especially albendazole (ABZ), due to its broad-spectrum, high antiparasitic efficacy, and low cost [4]. This drug is metabolized into two major metabolites, albendazole sulfoxide (ABZSO) (active) and albendazole sulfone (ABZSO_2_) (inactive) [5]. All benzimidazoles bind to parasite β tubulin, disrupting microtubule formation and all microtubule-based processes in the parasites, resulting in worm death [6]. The activity of these drugs depends on their ability to reach high and sustained concentrations over time at the site of the parasite [7], achieving the optimal activity when the target parasite is exposed to effective drug concentrations for a prolonged period of time. Nevertheless, their main disadvantage of benzimidazoles is related to their low water solubility and slow dissolution rate, which means that ABZ can only be administered orally. In ruminants, the acidic pH of the abomasum improves its dissolution and absorption in the small intestine [8].

Due to its wide use, ABZ is a drug for which plasma concentrations continue to be determined in man and various animal species, after its administration under different conditions or with other compounds [9]. Thus, different analytical methods have been developed to determine and quantify ABZ and metabolites, including the use of high-performance liquid chromatography with ultraviolet detection (HPLC-UV) [10] or diode array detection (HPLC-DAD) [11], micellar liquid chromatography-diode array detection (MLC-DAD) [12], and liquid chromatography-tandem mass spectrometry (LC-MS/MS) [13]. These methods have allowed the analysis of samples from different animal species, including the plasma of sheep and goats [14], laying hens [15], dogs [16], and cattle [17], as well as fecal samples of donkeys [18]. In some of them, specific instrumentation was required, not always available in standard laboratories [13,17,19,20,21]. In other cases, liquid-liquid extraction was carried out previously [14,16,18,22] or chromatograms were long [10,14,15,16,17,18,20,21,22,23,24]. In addition, none of them were carried out in accordance with the guidelines of the European Medicines Agency [25].

Taking into account all these considerations, it seems necessary to develop and validate a rapid, simple, and efficient bioanalytical method to determine and quantify ABZ and its metabolites. The method has been performed in cattle plasma, as this drug is commonly used in this animal species. Thus, the aim of this study was to carry out and validate an easy-to-use, fast, and relatively inexpensive HPLC method for ABZ determination and quantification in cattle plasma using an appropriate solid phase extraction procedure (SPE).

## 2. Results and Discussion

### 2.1. Selectivity

**Figure 1:**
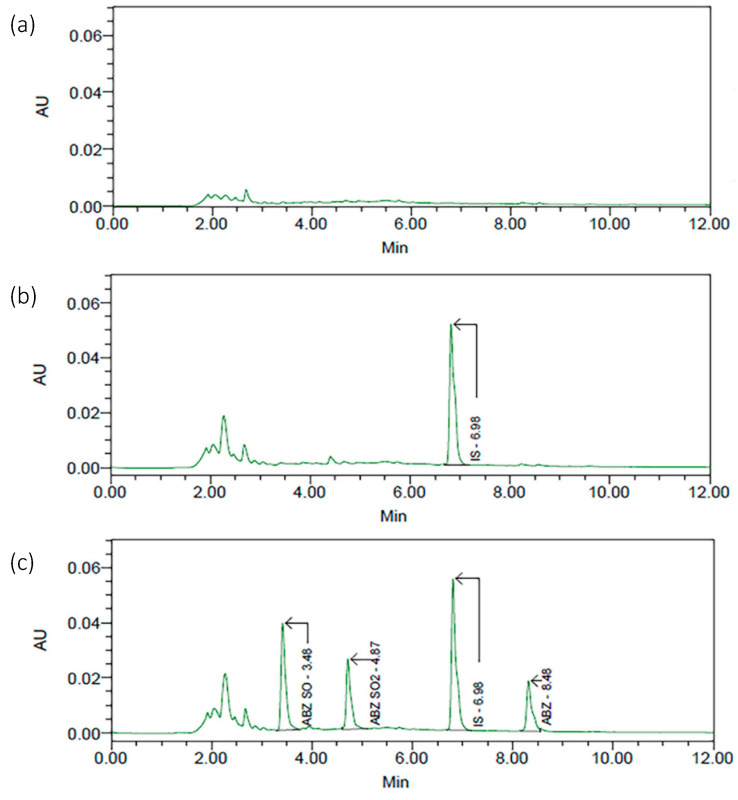
Chromatogram obtained from (**a**) blank plasma; (**b**) a spiked plasma sample with IS (oxibendazole); (**c**) from a spiked plasma sample with ABZ, ABZSO, ABZSO_2_, and IS.

No interfering peaks from endogenous components were detected at the retention times of ABZ, ABZSO, ABZSO_2_, and internal standard (IS) in the blank samples. Mean retention times were 3.5 min (ABZSO), 4.9 min (ABZSO_2_), 7.0 min (IS), and 8.5 min (ABZ) (Figure 1).

### 2.2. Specificity

As described below, no significant interference at the retention times of the analytes was found in the chromatograms of plasma blank samples.

### 2.3. Matrix Effect

It was assessed by analyzing three replicates of QC2 (0.075 µg/mL) and QC4 (1.5 µg/mL). Each sample was prepared from the matrix of each of the 6 different lots. No alteration in the analyte response was found due to interfering or unidentified compounds in the sample matrix. Moreover, accuracy and precision were within the criteria established in each individual lot.

### 2.4. Calibration Curve and Range

Calibration curves for ABZ, ABZSO, and ABZSO_2_ were defined by plotting the peak area ratio of each analyte to the IS vs. the concentration of the corresponding analyte. Calibration curves exhibited a linear relationship between the nominal analyte concentration and the analytical response obtained. Standard solutions used were within the range 0.025–2 µg/mL. Calibration curve parameters are summarized in Table 1 and were based on three independent runs of the three compounds (ABZ, ABZSO, and ABZSO_2_). Good linearity was evidenced, and high coefficients of determination were obtained (R^2^ > 0.999) (Figure S1 in the Supplementary Materials).

Back-calculated recoveries of each calibration concentration level were established from the calibration curve. All samples of each calibration curve were within ±20% of the nominal concentration at the LLOQ, and ±15% at all the other levels. Mean recovery and LOD of ABZ, ABZSO, and ABZSO_2_ were also calculated (Table 2), ranging from 100.0% in ABZSO_2_ to 101.6% in ABZ. Moreover, the LOD was 0.006 µg/mL for ABZ, 0.008 µg/mL for ABZSO, and 0.007 µg/mL for ABZSO_2_.

### 2.5. Accuracy and Precision

Table 3 details the accuracy and precision data of the method. Mean values of within-run and between-run accuracy for the four QC sample levels were in the range of 82.8–112.7% for ABZ, 85.3–117.7% for ABZSO, and 93.3–114.1% for ABZSO_2_**.** It is seen that the values obtained comply with the EMA criteria [25].

Regarding precision (% CV), the within-run and between-run precision (CVs for four QC levels) varied from 1.2% to 14.5% for ABZ, 0.8% to 15.1% for ABZSO, and 0.4% to 10.7%, and did not exceed the value of 15% (20% at the LLOQ). Consequently, our method met the accuracy and precision criteria described in the EMA guideline.

### 2.6. Carry-Over

No residual analyte was detected from a preceding sample that remained in the analytical instrument when blank samples were injected after the highest concentration calibration sample (2 µg/mL) and the highest quality control (ULOQ, 1.5 µg/mL) in each run.

### 2.7. Stability

ABZ, ABZSO, and ABZSO_2_ concentrations were calculated by using the calibration curves of the day of analysis. All the samples fulfilled the requirements of the EMA guideline, with accuracy ranging from 92.7 to 114.4%, and precision from 0.02 to 9.1% (Table 4).

### 2.8. Robustness

The method′s robustness was determined by analysis of samples under a variety of conditions, with small conscious changes in wavelength (±2 nm), flow rate (±0.2 mL/min), and pH of the mobile phase (±0.2). Throughout robustness testing, a solution at a concentration level of 1 μg/mL ABZ, ABZSO, ABZSO_2_, and IS was used. The results obtained showed that % RSD was less than 5%, confirming the robustness and reliability of the developed analytical method.

### 2.9. Method Application

**Figure 2:**
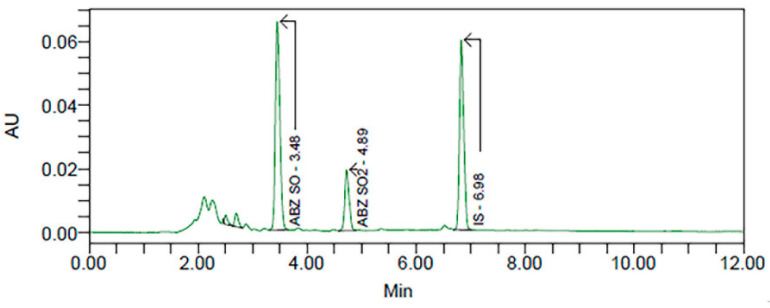
Chromatogram obtained from a calf plasma sample after ABZ oral administration (7.5 mg/kg).

The applicability of the method was evaluated by analyzing plasma samples in duplicate obtained from one healthy calf treated with ABZ at the oral dose of 7.5 mg/kg. Five samples were collected at different time points. The same retention times were obtained for ABZSO and ABZSO_2_ in plasma samples from the animal used to demonstrate the applicability of the method. Moreover, no potentially interfering peaks at the retention times of ABZ and metabolites were observed in the analyzed samples. Figure 2 shows a chromatogram with the three compounds detected in plasma (ABZSO, ABZSO_2_, and IS). No ABZ was detected in any plasma sample, which may be related to its extensive first-pass hepatic metabolism [26]. Thus, ABZSO and ABZSO_2_ were the only compounds recovered after ABZ oral administration, as reported in other studies [27]. Moreover, Table 5 details plasma concentrations of both ABZ metabolites determined in this animal.

The results obtained confirmed that the HPLC-PDA method developed was suitable for the determination of ABZ and its metabolites. We have shown that it fulfills the requirements of the EMA Guideline on Bioanalytical Method Validation [25], and it is a reliable analytical approach to be employed in pharmacokinetic studies.

It should be noted that one of the main challenges was to optimize the HPLC conditions to simultaneously quantify three compounds (ABZ, ABZSO, and ABZSO_2_) in a single method, saving both reagents and time.

The chromatographic separation method is simple and has a relatively short run time of 12 min. In addition, it can determine very low amounts of the analyte, which allows us to use small samples. The procedure is standardized and automated in order to serve as a basis for the validation of other drugs with similar chemical structures.

## 3. Materials and Methods

### 3.1. Animals and Experimental Procedure

Six healthy female Friesian calves of 4–5 months weighing 70–85 kg were used in this study. It was conducted under field conditions at a Spanish commercial farm located in the province of Leon. Calves were previously isolated from the rest of the animals and maintained under daily veterinary supervision. They had a one-week acclimatization period before the trial started, to provide accommodation to their new environment. Their diet consisted of a growing feed twice a day (daily ration 3–4 kg) with hay and straw *ad libitum* and free access to water. None of them had been medicated within the month prior to the study, and they were deemed healthy based on physical examination developed by a veterinarian prior to the start of the trial. This study was approved and reviewed by the Institutional Animal Care and Use Committee at the University of León (OEBA-ULE_015-2023).

A temporary indwelling catheter (Vasocan^®®^ 14G, Braun, Vetcare SA, Barcelona, Spain) was inserted into the left jugular vein just before blood collection (100 mL/animal). Blood was collected into heparinized tubes (sodium heparine). The catheter was removed once collection was completed. Blood was immediately centrifuged at 1500 rpm for 20 min, and plasma was stored at −20 °C until analysis.

### 3.2. Chemicals and Reagents

Pure reference standards of ABZ (analytical standard, ≥98%), ABZSO (Vetranal, analytical standard, ≥99%), ABZSO_2_ (Vetranal, analytical standard, ≥99%), and oxibendazole (internal standard, IS; Vetranal, analytical standard, ≥98%) were acquired from Sigma-Aldrich (Merck, Darmstadt, Germany). All solvents and reagents employed for drug extraction and analysis were HPLC grade: methanol (Li-Chrosolv Merck, Madrid, Spain), acetonitrile (HiPerSolv CHROMANORM, Radnor, PA, USA), ammonium acetate (VWR Chemicals, Leuven, Belgium), sodium hydroxide 1N (Panreac Quimica S.A., Barcelona, Spain), and hydrochloric acid 1M (Analyticals Carlo Erba, Milano, Italy). Ultrapure water (18.2 MΩ·cm resistivity at 25 °C; <5 ppb total organic carbon) was obtained with a Millipore Milli-Q gradient water purification system.

### 3.3. Analytical Procedure

#### 3.3.1. Preparation of Stock, Calibration, and Quality Control Working Solutions

Stock solutions, calibration, and quality control working solutions were prepared daily. Initially, 4 stock solutions (2 mg/mL) were prepared in HPLC grade water by adding 20 mg of each compound (ABZ, ABZSO, ABZSO_2_, and IS) independently in 1 mL of methanol. The first three stock solutions (ABZ, ABZSO, and ABZSO_2_) were then combined in one (200 µg/mL each), whereas an independent solution was prepared for IS (200 µg/mL).

Calibration working solutions containing ABZ and metabolites at six different concentration levels (0.25, 0.5, 1, 5, 10, 20 µg/mL) and IS (10 µg/mL) were made by diluting an adequate volume of each stock solution in 10 mL of HPLC grade water.

Quality control working solutions (QC) were prepared at 4 distinct concentration levels: QC1, the lower limit of quantification (LLOQ, 0.25 µg/mL); QC2, three times the LLOQ (LOW, 0.75 µg/mL); QC3, between 30 and 50% of the calibration curve range (MED, 7 µg/mL), and QC4, at least 75% of the upper calibration curve range (ULOQ, 15 µg/mL).

#### 3.3.2. Preparation of Analysis Samples

All samples were completely defrosted at room temperature:

- Blank samples: biological matrix (1 mL) without ABZ, ABZSO, and ABZSO_2_.
- Zero samples: blank sample (0.9 mL) spiked with 0.1 mL of IS.
- Calibration standards, prepared by spiking 0.9 mL matrix with 0.1 mL of each calibration working solution. Thus, calibration sample concentrations were: 0.025, 0.05, 0.1, 0.5, 1, and 2 µg/mL for ABZ, ABZSO, and ABZSO_2_, and 1 µg/mL for IS.
- Quality control samples were prepared in plasma (0.9 mL) at concentrations of 0.025, 0.075, 0.7, and 1.5 µg/mL for ABZ, ABZSO, and ABZSO_2_, and 1 µg/mL for IS.

#### 3.3.3. Extraction Method

A solid-phase extraction (SPE) was conducted to determine ABZ and metabolites from plasma samples. Oasis HLB 3 cc 60 mg cartridges (Waters Corporation, Milford, MA, USA) were used. They were conditioned with 1 mL of methanol and 1 mL of ultrapure water. Subsequently, 1 mL of plasma was added, and the cartridges were washed with 3 mL of water and dried with air for 5 min. The cartridge was then eluted with 2 mL of methanol. The eluate was evaporated to dryness under a gentle nitrogen stream. Finally, the residue was reconstituted with 0.25 mL mobile phase, and 50 µL was injected into the chromatographic system.

#### 3.3.4. HPLC System and Conditions

Samples were analyzed by reverse-phase high-performance liquid chromatography (HPLC) in a Waters Alliance e2695 equipped with a photodiode array detector (PDA) (model 2998) (Waters Corporation, Milford, MA, USA). Separation was performed by using an XBridge C18 column (4.6 mm × 250 mm, 5 µm, Waters).

The mobile phase consisted of acetonitrile:ammonium acetate buffer 0.025 M with pH adjusted to 6.6 (200 µL HCl 1 M). Gradient elution was performed at a flow rate of 1.2 mL/min for 12 min. Gradient program changed from 27:73 to 50:50 for 5 min, maintained then for 4 min, dropped again to 27:73 in 1 min, and maintained for 2 min. The injection volume was 50 µL, and the PDA detector was set at 292 nm.

### 3.4. Validation of the Analytical Methodology

The developed method for plasma was validated according to validation procedures, parameters, and acceptance criteria established in the European Medicines Agency guideline EMA/CHMP/ICH/172948/2019 for the following parameters: Selectivity, specificity, matrix effect, calibration curve and range, accuracy, precision, carry-over, and stability [25].

#### 3.4.1. Selectivity

Selectivity was performed to assess the interference at the retention times of the three analytes and the IS. Six blank plasma samples from six different individuals were analyzed. This evaluation should demonstrate no significant response attributable to interfering components at the retention times of the analyte or the IS in the blank samples. These interfering responses should not be more than 20% of the analyte response and not more than 5% of the IS response, both in the LLOQ sample for each matrix.

#### 3.4.2. Specificity

Specificity was evaluated to detect and differentiate the analyte from other compounds, including those related. Responses detected and due to interfering components should not exceed 20% of the analyte response at the LLOQ and 5% of the IS response in the LLOQ sample.

#### 3.4.3. Matrix Effect

The matrix effect between different independent sources was evaluated. For each matrix lot, the accuracy should be within ±15% of the nominal concentration, and precision (% CV) should not be more than 15%.

#### 3.4.4. Calibration Curve and Range

The calibration range was defined by the LLOQ and the ULOQ. The LLOQ was assessed in blank samples spiked with the lowest quality control working solution (0.25 µg/mL), and the ULOQ (15 µg/mL) was 75% of the highest calibration standard (20 µg/mL). The calibration curve was generated with a blank sample, a zero sample, and six concentration levels from 0.025 to 2 µg/mL for ABZ, ABZSO, and ABZSO_2_. IS concentration was 1 µg/mL.

A simple regression model was employed to describe the concentration-response relationship. It was carried out on known concentrations of ABZSO and ABZSO_2_ against the ratio of the area of ABZ vs. IS.

These samples were analyzed in a minimum of 3 independent runs carried out in duplicate over 3 days. The determination coefficient (R^2^), the slope, and the intercept of the resulting calibration curves were determined. These parameters were calculated without both blank and zero samples.

#### 3.4.5. Accuracy and Precision

Both parameters were established by analyzing the QCs within each run (within-run) and in different runs (between-run). Accuracy and precision were evaluated with the same runs and data.

For the within-run 5 replicated measures at each QC concentration level in each run were used, whereas for the between-run precision and accuracy, each QC level was assessed 5 times in 3 analytical runs on 3 different days. Precision was expressed as the coefficient of variation (CV), and no concentration determined may exceed 15%, except LLOQ, which may not be higher than 20%. Regarding accuracy at each concentration level, it should be within ±15% of the nominal concentration, except at the LLOQ, which should be within ±20%.

#### 3.4.6. Carry-Over

It was tested in blank plasma samples injected after the highest calibration standard (2 µg/mL) and the ULOQ (1.5 µg/mL) in each run. The carryover in blank samples cannot exceed 20% of the analyte response at the LLOQ and 5% of the response for the IS.

#### 3.4.7. Stability

Stability was tested by evaluating QC2 and QC4 samples at different storage temperatures and over different periods of time. For each concentration assessed, there was a bulk sample divided into 3 aliquots, which were tested through three freeze-thaw cycles from −20 °C to room temperature.

#### 3.4.8. Robustness

The robustness of this analytical method was investigated by applying minor and purposeful changes to the chromatographic conditions, which included variation in the flow rate, pH of the mobile phase, and detection wavelength, while keeping the other conditions unchanged. Robustness of the present method was determined in terms of % RSD.

### 3.5. Method Application

To verify the applicability of the method developed in the clinical practice, ABZ and metabolite concentrations were measured in plasma samples from one healthy 4-month-old Friesian female weighing 82 kg. For this purpose, ABZ (Albendex 100 mg/mL^®®^, S.P. Veterinaria, S.A., Tarragona, Spain) was orally administered with a dosing device at a dose of 7.5 mg/kg.

Blood samples were collected by venipuncture from both jugular veins alternately into heparinized tubes (6 mL Vacutainer, BD, Plymouth, UK) just before administration and at 0.2, 0.4, 1, 1.5, and 2 h. Samples were immediately centrifuged at 1500 rpm for 20 min after collection and stored at −20 °C until analysis. IS was added at the time of plasma analysis. The animal procedure had also been approved by the Ethics Committee of the University of Leon (OEBA-ULE-015-2023).

### 3.6. Data Analysis

The HPLC Empower 3 software (Waters Corporation, Milford, MA, USA) was employed for data acquisition and processing. A descriptive statistic (mean, standard deviation, and percentages) was performed with SPSS Statistical Software v. 26.0 (IBM Corporation, Armonk, NY, USA).

## 4. Conclusions

The development and validation of an HPLC quantification method for ABZ and its metabolites has been shown. The method is sensitive, accurate, and precise, and three related analytes may be determined simultaneously. It is also cost- and time-saving, sample preparation is simple, and the HPLC technique followed may be used in laboratories with standard equipment. The clinical applicability of our method was also demonstrated.

## Figures and Tables

**Table 1:** Results of three independent calibration curves for ABZ, ABZSO, and ABZSO_2_.

	Calibration Curve 1	R_2_	Calibration Curve 2	R^2^	Calibration Curve 3	R^2^
ABZ	y = 0.7839 (±5.514·10^−3^) x + 0.0055 (±5.164·10^−3^)	0.9995	y = 0.9165 (±5.849·10^−3^) x + 0.0276 (±5.478·10^−3^)	0.9995	y = 0.9123 (±5.348·10^−3^) x + 0.0263 (±5.009·10^−3^)	0.9991
ABZSO	Y = 1.4318 (±7.733·10^−3^) x + 0.0124 (±7.242·10^−3^)	0.9997	y = 1.3006 (±4.803·10^−3^) x − 0.0003 (±4.499·10^−3^)	0.9998	y = 1.2974 (±4.593·10^−3^) x + 0.0153 (±4.304·10^−3^)	0.9999
ABZSO_2_	y = 0.943 (±4.467·10^−3^) x + 0.0068 (±4.184·10^−3^)	0.9998	y = 1.3176 (±6.069·10^−3^) x + 0.0256 (±5.684·10^−3^)	0.9998	y = 1.3155 (±3.944·10^−3^) x + 0.0271 (±3.694·10^−3^)	0.9999

Standard error of the slope and the intercept of the calibration curve equations are given in brackets.

**Table 2:** LLOQ, LOD, and recovery for ABZ, ABZSO, and ABZSO_2_.

	LLOQ (µg/mL)	LOD (µg/mL)	$\mathrm{Recovery}\;(\%)\;(\bar{x}$ ± SD)
ABZ	0.025	0.006	101.6 ± 0.7
ABZSO	0.025	0.008	100.4 ± 1.3
ABZSO_2_	0.025	0.007	100.0 ± 1.4

$\bar{x}$: mean; SD: standard deviation.

**Table 3:** Accuracy and precision results.

	ABZ	ABZSO	ABZSO_2_
Accuracy (%)			
Within-run			
QC1	82.8–105.9	85.3–117.7	94.8–114.1
QC2	98.0–112.7	96.2–111.0	93.3–104.6
QC3	98.1–100.5	95.3–103.7	94.3–102.9
QC4	99.4–104.1	98.7–101.9	97.6–100.3
Between-run			
QC1	91.5	100.0	107.4
QC2	107.5	106.1	98.1
QC3	99.0	100.7	99.5
QC4	101.1	100.4	99.4
Precision (%)			
Within-run			
QC1	3.4–11.7	2.8–10.3	4.2–10.7
QC2	2.7–4.2	3.1–7.0	2.8–5.5
QC3	1.2–5.1	1.7–3.6	1.2–3.3
QC4	1.5–5.8	0.8–4.8	0.4–1.5
Between-run			
QC1	14.5	15.1	10.6
QC2	7.3	8.5	7.4
QC3	3.2	4.6	4.4
QC4	4.0	3.1	1.7

**Table 4:** Stability results of QC samples for ABZ, ABZSO, and ABZSO_2_.

T^a^ (°C)		Time	ABZ		ABZSO		ABZSO_2_	
			Precision (%)	Accuracy (%)	Precision (%)	Accuracy (%)	Precision (%)	Accuracy (%)
QC2	25	24 h	8.5	106.6	5.4	92.7	9.1	105.2
		24 h after extraction	8.8	109.3	6.2	98.4	0.8	114.4
	4	24 h	6.1	107.6	1.2	99.6	1.3	99.6
		24 h after extraction	8.9	103.4	5.2	99.0	7.6	104.3
	−20	72 h	6.8	105.1	4.5	106.0	5.3	110.4
		1 week	0.7	114.2	6.7	99.0	1.0	107.5
		1 month	2.7	113.2	4.5	100.4	2.2	110.4
		4 months	1.1	112.2	1.0	96.6	3.8	109.3
		6 months	3.5	108.5	3.6	95.4	2.9	108.2
QC4	25	24 h	0.4	99.1	0.02	97.9	1.2	97.2
		24 h after extraction	0.3	99.9	1.8	100.2	1.7	99.1
	4	24 h	1.1	99.5	0.2	97.4	2.0	99.9
		24 h after extraction	1.2	99.4	0.6	98.2	0.04	97.2
	−20	72 h	0.7	101.9	2.7	100.3	2.3	99.1
		1 week	1.2	98.6	0.2	98.2	1.3	97.6
		1 month	2.3	100.4	0.3	98.2	1.5	98.4
		4 months	1.7	100.1	1.2	99.7	1.2	98.3
		6 months	0.2	99.6	0.3	99.1	1.2	99.3

**Table 5:** Plasma sample concentrations for ABZ, ABZSO, and ABZSO_2_ from one healthy calf after ABZ oral administration (7.5 mg/kg).

Time (h)	ABZ (µg/mL)	ABZSO (µg/mL)	ABZSO_2_ (µg/mL)
0.25	ND	0.026	ND
0.5	ND	0.076	0.055
1	ND	0.335	0.108
1.5	ND	0.743	0.165
2	ND	1.3	0.240

ND: not detected.

## Data Availability

The original contributions presented in this study are included in the article. Further inquiries can be directed to the corresponding author.

## Supplementary Materials

The following supporting information can be downloaded at: https://www.mdpi.com/article/10.3390/molecules30092039/s1, Figure S1: Calibration curves for ABZ; Figure S2: Calibration curves for ABZSO; Figure S3: Calibration curves for ABZSO_2_.
