# Supplementary material for: Analytical Method for the Simultaneous Determination of Albendazole and Metabolites Using HPLC-PDA: A Validation Study
Source: Molecules. 2025 May 3;30(9):2039. doi: 10.3390/molecules30092039 (PMC12073153; doi:10.3390/molecules30092039)
Supplement: Supplementary file 1 [file molecules-30-02039-s001.zip › molecules-3592108-supplementary.pdf]

Supplementary material.

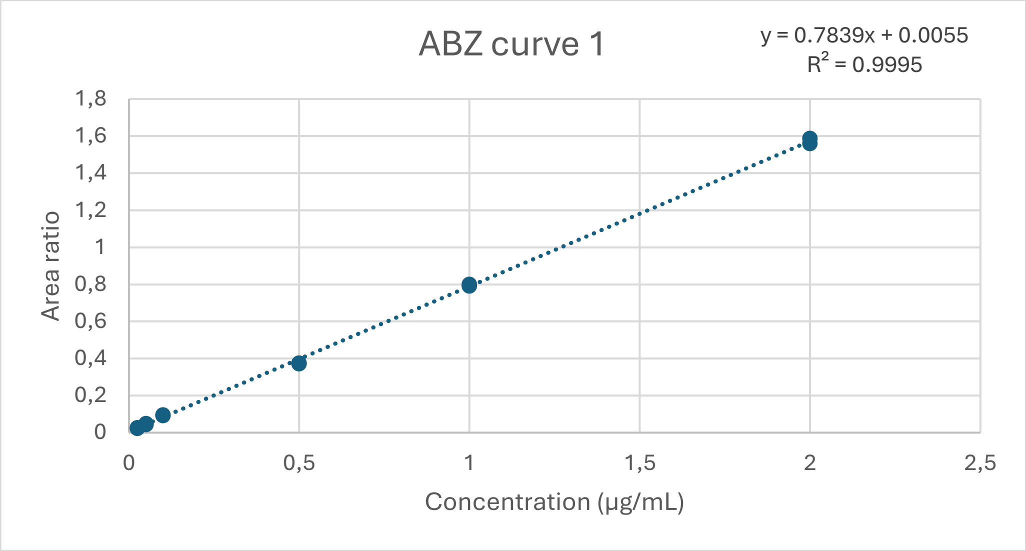

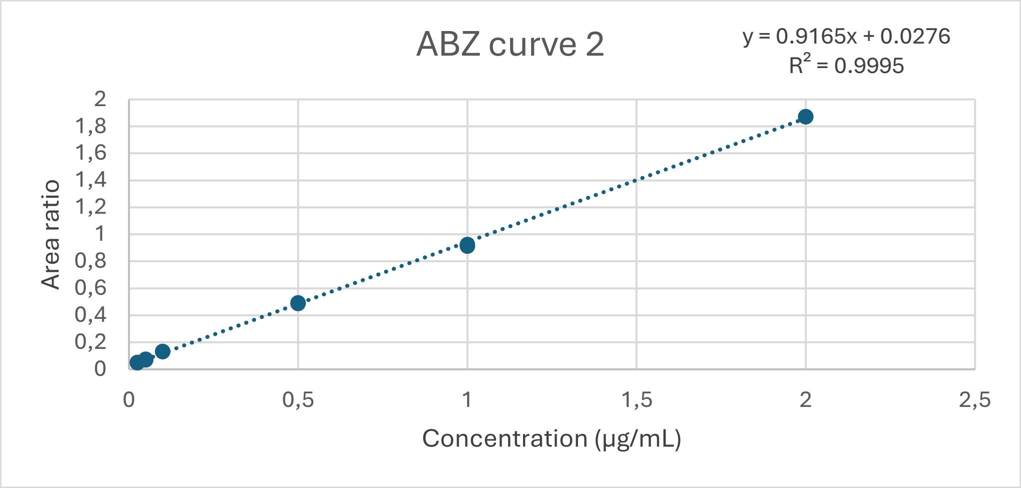

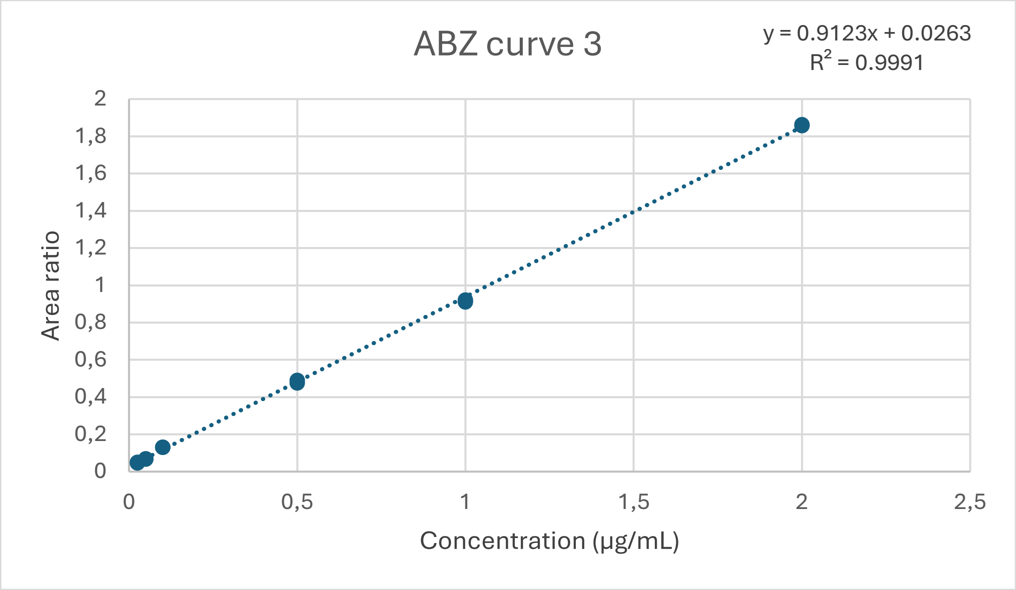

Figure S1. Calibration curves for ABZ.

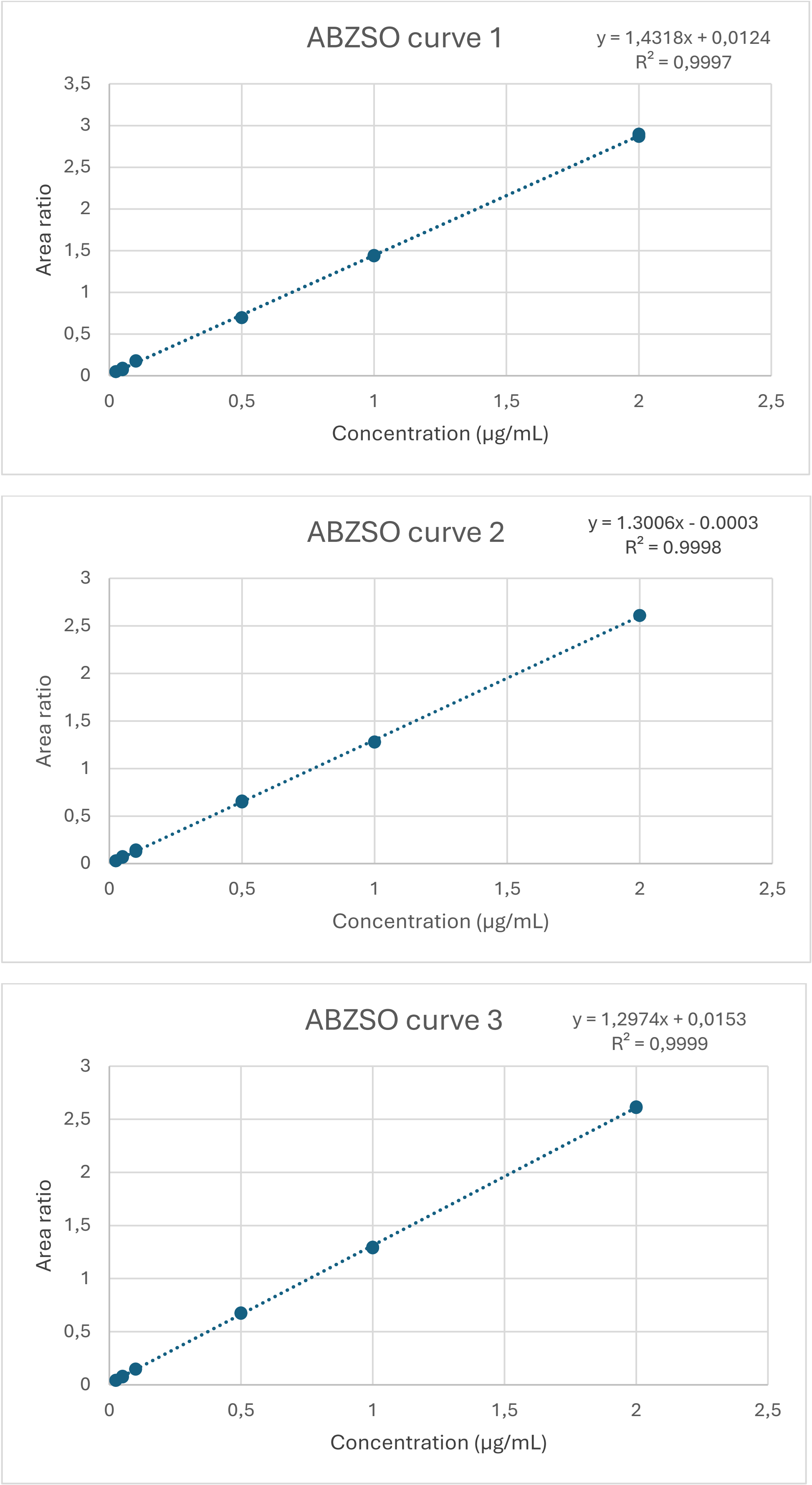

Figure S2. Calibration curves for ABZSO.

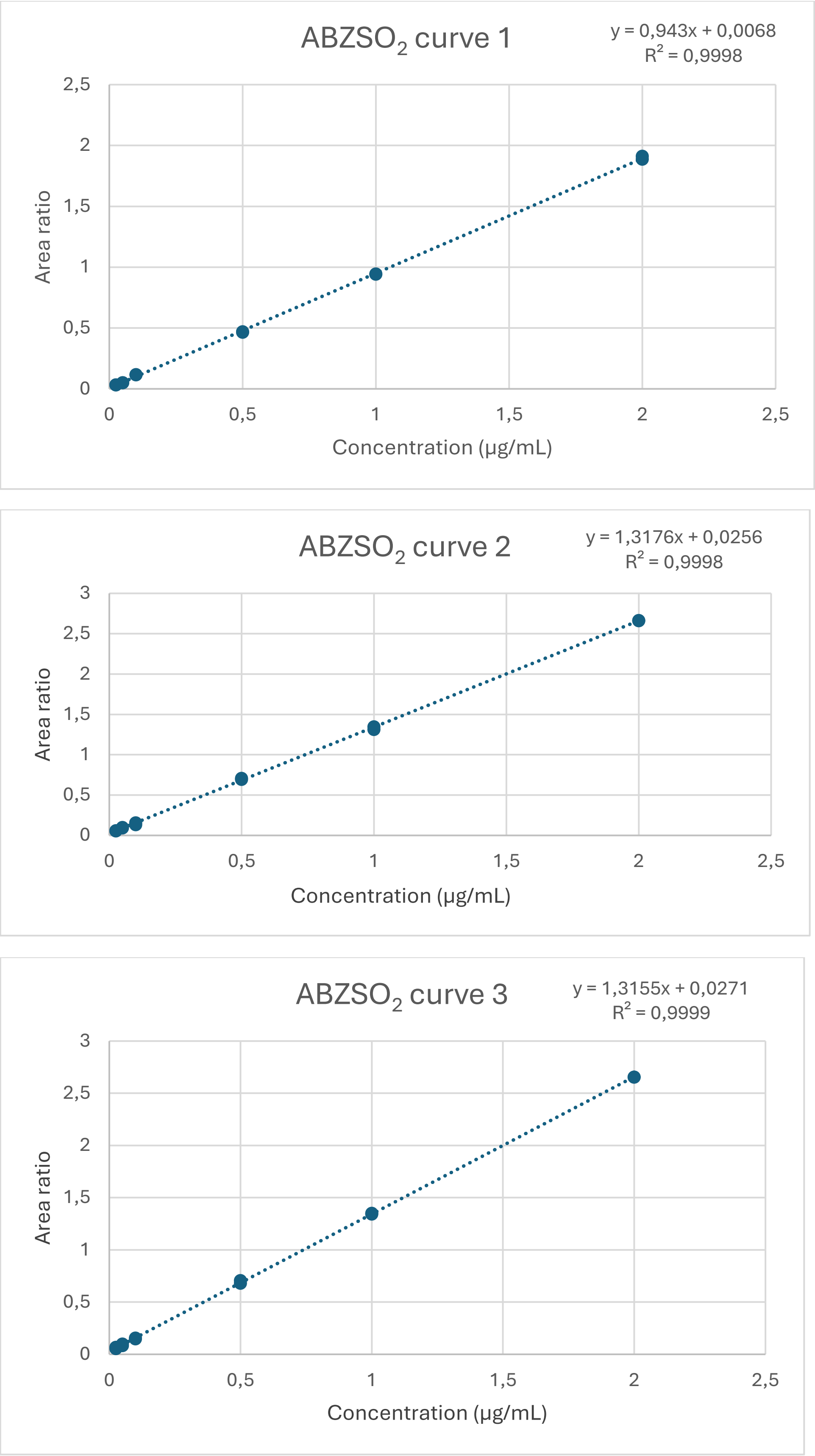

Figure S3. Calibration curves for $ABZSO_2$.
